# Berbamine Promotes the Repair of Lower Limb Muscle Damage in Chronic Limb-Threatening Ischemia by Inhibiting Local Inflammation and NF-κB Nuclear Translocation

**DOI:** 10.3390/ph17121583

**Published:** 2024-11-25

**Authors:** Lei Zheng, Biao Zhao, Zhenxi Zhang, Yutong Liu, Yingying Zhang, Jing Cai, Tong Qiao

**Affiliations:** 1 Department of Vascular Surgery, Nanjing Drum Tower Hospital, Affiliated Hospital of Medical School, Nanjing University, Zhongshan Road 321, Nanjing 210008, China; df21350078@smail.nju.edu.cn (L.Z.); 622023350106@smail.nju.edu.cn (B.Z.); 602023350088@smail.nju.edu.cn (Z.Z.); dg1935015@smail.nju.edu.cn (Y.L.); dg1735039@smail.nju.edu.cn (J.C.); 2 Department of Neurosurgery, Nanjing Drum Tower Hospital, The Affiliated Hospital of Nanjing University Medical School, Zhongshan Road 321, Nanjing 210008, China; mf20350076@smail.nju.edu.cn

**Keywords:** berbamine, limb ischemia, inflammation, nuclear factor kappa-B, apoptosis

## Abstract

**Background/Objectives**: Chronic Limb-Threatening Ischemia (CLTI) is a chronic limb ischemic disease caused by vascular lesions, characterized by pain, ulcers, and gangrene, which can be life-threatening in severe cases. The objective of this study is to explore whether Berbamine (BBM) can protect against and repair ischemic muscle tissue in the lower limbs; **Methods**: Using a mouse hindlimb ischemia (HLI) model, 36 C57BL6 mice were divided into sham, HLI, and HLI+BBM treatment groups. **Results**: Our findings indicate that BBM can restore motor function and muscle tissue pathology in mice, potentially by inhibiting the nuclear translocation of nuclear factor kappa-B (NF-κB), thereby alleviating tissue inflammation caused by chronic ischemia, reducing muscle cell apoptosis, inhibiting M1 macrophage polarization, and promoting angiogenesis. **Conclusions**: Our research suggests that BBM has the potential to protect against ischemic damage in lower limb muscle tissue, providing a new approach to the treatment of CLTI.

## 1. Introduction

Chronic Limb-Threatening Ischemia (CLTI) is a severe vascular disease primarily characterized by inadequate blood flow to the lower limbs, leading to symptoms such as pain, ulcers, and gangrene [1,2,3]. Currently, approximately 40 million people worldwide are afflicted with CLTI, and the incidence rate is rising annually due to factors such as population aging and the increasing prevalence of chronic diseases like diabetes [1]. Therefore, delving into the pathogenesis of CLTI and searching for effective treatment methods have become significant research topics in modern medicine.

The primary treatment objectives include promoting arteriogenesis or angiogenesis, correcting the pathological changes in ischemic leg muscles, and limiting the progression of atherosclerotic thrombosis in the arteries supplying the lower limbs, as well as surgical interventions [4,5]. Berbamine (BBM) is a natural compound extracted from the traditional Chinese medicine *Berberis amurensis* and possesses a variety of biological activities [6]. It has the function of inhibiting Ca^2+^/calmodulin-dependent protein kinase II γ (CaMKII γ) and nuclear factor kappa-B (NF-κB) [7,8,9,10,11]. Previous studies have found that BBM has potential therapeutic effects on prostate cancer [8], colon cancer [12], and leukemia [10] by inhibiting the NF-κB signaling pathway. Additionally, it shows therapeutic value in non-cancerous diseases such as liver injury [13], diabetes [14], and ischemia–reperfusion injury in the heart and brain [15,16]. BBM also has the ability to alleviate bone loss caused by excessive osteoclast activity [17]. The NF-κB signaling pathway is markedly elevated in the muscle tissue of patients suffering from peripheral arterial disease (PAD), correlating strongly with inflammation induced by ischemia [18]. Hence, we hypothesize that BBM could be employed to treat muscle lesions in patients with PAD.

In our study, we constructed a classical hindlimb ischemia model. This was completed in adult male C57BL/6 mice. Our goal was to investigate whether BBM could play a potential protective role in hindlimb ischemia. Additionally, we aimed to illustrate its pathophysiological and pharmacological mechanisms.

## 2. Results

### 2.1. BBM Improves Hind Limb Ischemia in Mice by Enhancing Motor Function and Skeletal Muscle Regeneration

**Figure 1:**
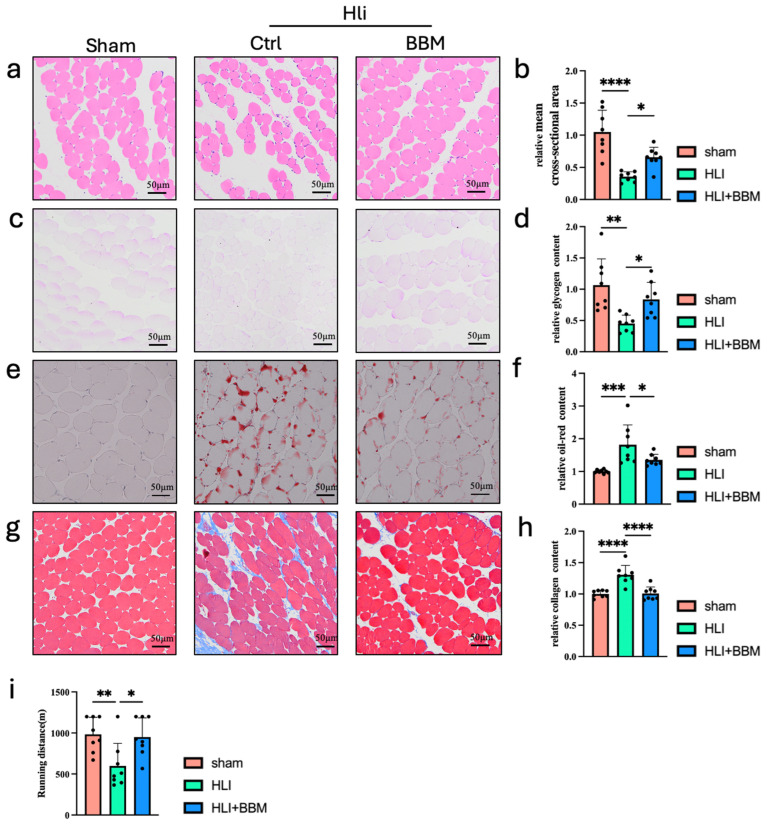
BBM enhances motor function and ameliorates muscle tissue pathology in mice with hindlimb ischemia (HLI). (**a**) Representative images of gastrocnemius muscles, measured by H&E staining (transverse section). (**b**) Quantification of the mean cross-sectional area of skeletal muscle HE staining, n = 8 per group. (**c**,**d**) Representative images of PAS staining and quantification of gastrocnemius muscles, transverse section, n = 8 per group. (**e**,**f**) Representative images of oil red O staining and quantification of gastrocnemius muscles, transverse section, n = 8 per group. (**g**,**h**) Representative images of Masson staining and quantification of gastrocnemius muscles, transverse section, n = 8 per group. (**i**) The endurance running distance of mice in each group over a fixed time, n = 8 per group. Data are expressed as mean ± SEM. * *p* < 0.05, ** *p* < 0.01, *** *p* < 0.001, **** *p* < 0.0001.

To investigate whether BBM could mitigate the hind limb muscle damage induced by ischemia, we obtained muscle samples from sham mice and from mice with hind limb ischemia that were administered BBM for analysis. Hematoxylin and eosin (HE) staining demonstrated that the muscle cross-sectional area distribution and average cross-sectional area were substantially increased in the BBM-treated group (Figure 1a,b). Ischemic skeletal muscle tissue is characterized by glycogen depletion, and Periodic Acid–Schiff (PAS) staining revealed that hind limb ischemia resulted in a significant reduction in muscle glycogen, which was notably attenuated by BBM treatment (Figure 1c,d). Muscle regeneration is compromised when the number or function of muscle satellite cells is abnormal, leading to the replacement of muscle fibers with ectopic tissues such as fat and fibrous tissue [19]. To evaluate the extent of fat and fibrous tissue deposition in ischemic skeletal muscle, we performed oil red O staining (Figure 1e,f) and Masson staining (Figure 1g,h) on sections of ischemic gastrocnemius muscle. Our research indicates that BBM effectively reduces the accumulation of lipids and collagen in muscle tissue affected by hind limb ischemia. Through endurance running experiments, we found that BBM significantly enhanced the exercise endurance of mice (Figure 1i). These findings suggest that BBM can markedly improve the reduced motor function and skeletal muscle damage associated with hind limb ischemia in mice.

### 2.2. BBM Promotes Angiogenesis in the Ischemic Hind Limbs of Mice

**Figure 2:**
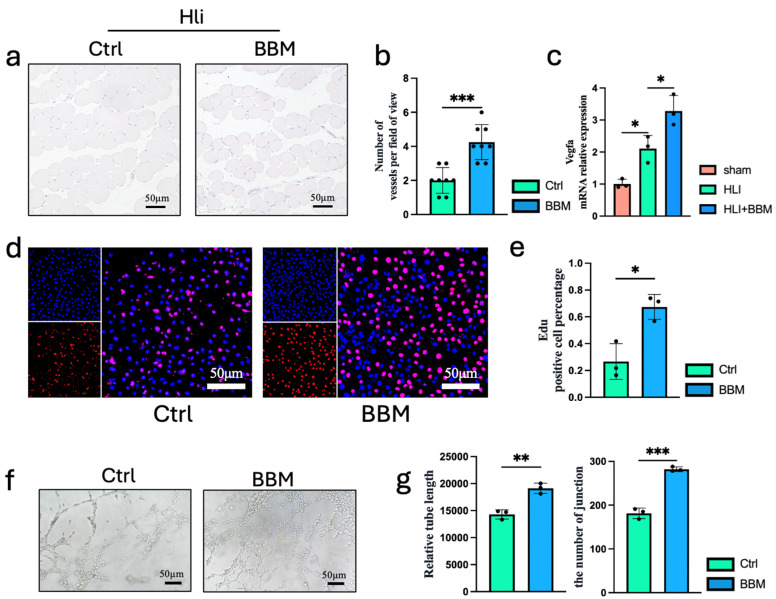
BBM promotes angiogenesis and skeletal muscle regeneration in the ischemic hind limbs of mice. (**a**,**b**) Representative immunohistochemistry images and quantification of CD31 in gastrocnemius muscles transverse sections, n = 8 per group. (**c**) The mRNA expression of *VEGFA* in gastrocnemius muscles examined by qPCR, n = 3 per group. (**d**,**e**) Representative immunofluorescence images and quantification of Edu in HUVECs, n = 3 per group. (**f**,**g**) Representative images and quantification of relative tube length and number of junctions in angiogenesis test, n = 3 per group. Data are expressed as mean ± SEM. * *p* < 0.05, ** *p* < 0.01, *** *p* < 0.001.

Angiogenesis is a vital compensatory mechanism in response to limb ischemia and a key process in the repair of ischemic damage. To assess the impact of BBM on vascular distribution in the ischemic hind limbs, we employed CD31 immunohistochemistry and observed that BBM markedly increased the formation of new blood vessels in the ischemic hind limbs (Figure 2a,b). Furthermore, qPCR analysis revealed that BBM upregulated the expression of *Vascular Endothelial Growth Factor A* (*VEGFA*) at the transcriptional level in the context of hind limb ischemia (Figure 2c). By collecting the culture supernatant from peritoneal macrophages cultured in a hypoxia chamber and applying it to HUVECs, we found that the angiogenic potential of HUVECs was significantly enhanced in the BBM-treated group (Figure 2d–g). The HUVECs in the BBM-treated group exhibited significantly higher proliferation and angiogenic capabilities compared to the control group. The restoration of skeletal muscle damage relies on the activation, proliferation, and myogenic differentiation of muscle satellite cells. These findings suggest that BBM promotes angiogenesis and enhances blood circulation in the ischemic hind limbs.

### 2.3. BBM Inhibits M1 Polarization of Macrophages in the Ischemic Hind Limbs of Mice

**Figure 3:**
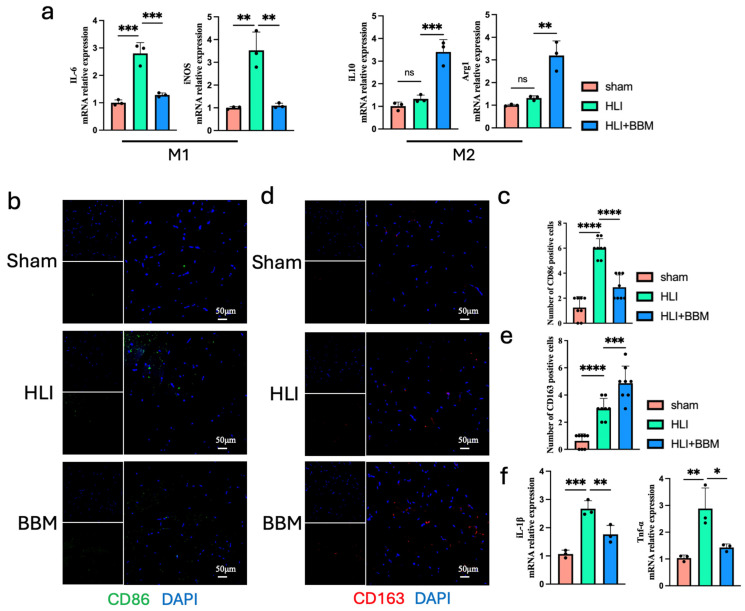
BBM inhibits M1 polarization of macrophages and inflammation in the ischemic hind limbs of mice. (**a**) The mRNA expression of *iNOS*, *IL-6*, *IL-10*, and *Arg1* in gastrocnemius muscles examined by qPCR, n = 3 per group. (**b**,**c**) Representative immunofluorescence images and quantification of CD86 in gastrocnemius muscles transverse sections, n = 8 per group. (**d**,**e**) Representative immunofluorescence images and quantification of CD163 in gastrocnemius muscles transverse sections, n = 8 per group. (**f**) mRNA expression level of *IL-1β* and *TNF-α* in the gastrocnemius muscles of mice, detected by qPCR, n = 3 per group. Data are expressed as mean ± SEM. * *p* < 0.05, ** *p* < 0.01, *** *p* < 0.001, **** *p* < 0.0001; ns: not significant.

Inflammation is closely associated with the development and progression of PAD and is related to poor prognosis in PAD patients [18,20,21,22]. By inhibiting M1 macrophage polarization, the secretion of inflammatory factors by macrophages can be suppressed [23]. To examine whether BBM could inhibit the polarization of M1-type macrophages induced by hindlimb ischemia, we performed qPCR experiments. The findings revealed that BBM significantly decreased the mRNA expression levels of *iNOS* and *IL-6*, which are indicative of M1 macrophage polarization, while it increased the mRNA expression of *IL-10* and *Arg1*, markers associated with M2-type macrophage polarization (Figure 3a). Immunofluorescence staining also demonstrated that the expression of CD86, a marker for M1 macrophages, was notably enhanced in the muscles of mice with hind limb ischemia, and this increase was markedly suppressed by BBM treatment (Figure 3b,c). Additionally, the expression of CD163, a marker for M2 macrophages, was significantly elevated in the muscle tissue of the BBM-treated group (Figure 3d,e). Through qPCR analysis, we also observed a significant increase in the mRNA levels of the inflammation-related genes Tnf-α and Il-1β (Figure 3f). These results indicate that BBM can inhibit M1 macrophage polarization and promote the shift towards an M2 phenotype, thereby dampening inflammation and fostering angiogenesis in the ischemic hind limbs of mice.

### 2.4. BBM Inhibits Apoptosis Induced by Hindlimb Ischemia in Mice

**Figure 4:**
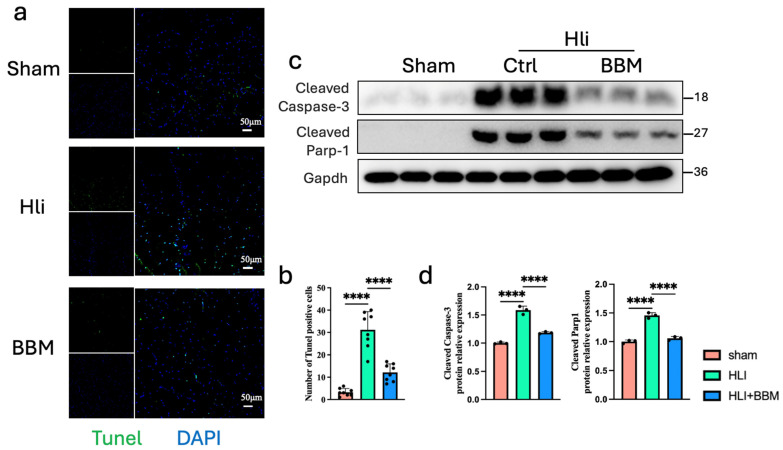
BBM inhibits apoptosis induced by hind limb ischemia in mice. (**a**,**b**) Representative immunofluorescence images and quantification of Tunel staining in gastrocnemius muscles transverse sections, n = 8 per group. (**c**,**d**) Representative immunoblot bands and quantitative analysis of cleaved-Caspase-3 and cleaved-Parp1 in gastrocnemius muscles, n = 3 per group. Data are expressed as mean ± SEM. **** *p* < 0.0001.

Apoptosis-related genes are significantly upregulated in the lower limb muscle tissue of PAD patients [18]. NF-κB possesses dual functions: it can both promote tissue inflammation, thereby exacerbating apoptosis, and facilitate the survival of tissue cells. However, NF-κB may play a more significant role in chronic inflammation resulting from ischemia and hypoxia. To assess whether BBM could inhibit the impact of hind limb ischemia on muscle tissue apoptosis in mice, we conducted TUNEL staining. The TUNEL staining revealed a substantial increase in the percentage of apoptotic cells within the ischemic muscle tissue, which was attenuated by BBM treatment (Figure 4a,b). Subsequently, we investigated the expression of apoptosis-related proteins via Western blot analysis. We observed a significant upregulation of caspase-3 and cleaved PARP1 in the hind limb ischemia model, and BBM significantly decreased the expression of these proteins in the ischemic muscle (Figure 4c,d). These findings suggest that BBM can reduce the apoptosis of muscle tissue induced by hind limb ischemia.

### 2.5. BBM Inhibits NF-κB Nuclear Translocation

**Figure 5:**
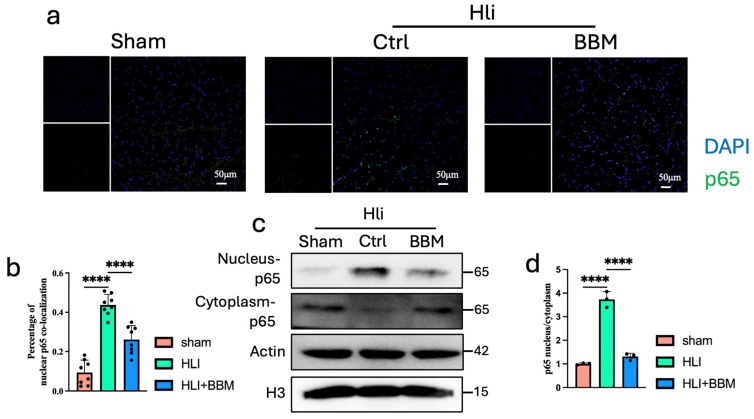
Berbamine inhibits NF-κB nuclear translocation. (**a**,**b**) nuclear translocation was determined by immunofluorescence staining for p65 (red) and DAPI staining for DNA (blue), n = 8 per group. (**c**,**d**) Nuclear p65 protein expression in gastrocnemius muscles was analyzed by western blot, n = 3 per group. Data are expressed as mean ± SEM. **** *p* < 0.0001.

Previous studies have found that the NF-κB signaling pathway is significantly upregulated in PAD tissues [18]. Furthermore, research has demonstrated that BBM can down-regulate IKKα and p-IκBα, subsequently inhibiting the nuclear translocation of NF-κB, which results in reduced expression of NF-κB downstream targets [7,10]. Moreover, the activation of the NF-κB signaling pathway is a crucial transcription factor that facilitates M1 polarization in macrophages [24]. NF-κB activation is mediated by its translocation into the nucleus, where it acts as a transcription factor. Initially, through immunofluorescence detection, we discovered that BBM can inhibit the nuclear translocation of NF-κB induced by hypoxia and ischemia in HLI tissue (Figure 5a,b). After isolating nuclear and cytoplasmic proteins separately and performing Western blot analysis, we observed a notable increase in the nuclear content of NF-κB protein within the ischemic tissue, and BBM was found to decrease the nuclear NF-κB protein levels (Figure 5c,d). These findings suggest that BBM protects against lower limb ischemia by suppressing the nuclear translocation of NF-κB.

## 3. Discussion

In this study, we discovered that BBM can rectify muscle tissue damage induced by hind limb ischemia by inhibiting the nuclear translocation of NF-κB. The primary research objectives of this paper encompass: (1) establishing that BBM can alleviate muscle damage caused by hind limb ischemia in mice; (2) demonstrating that BBM treatment reduces inflammation and apoptosis while promoting angiogenesis in muscle tissue impacted by hind limb ischemia; and (3) revealing that the anti-inflammatory mechanism of BBM is linked to its suppression of NF-κB nuclear translocation.

PAD is caused by factors such as atherosclerosis, leading to insufficient blood supply to the distal limbs. The main symptoms are lower limb pain, numbness, weakness, etc. Early symptoms include intermittent claudication, while late symptoms include ischemic rest pain, limb-end ischemic necrosis, ulcers, and gangrene, which may eventually lead to amputation or threaten life, a condition known as CLTI [1,2,3]. The main pathology of PAD includes microvascular dysfunction and a decrease in muscle volume and density [2]. In addition to surgical revascularization, the current main treatment methods for PAD include Cilostazol, lipid-lowering medications, and exercise therapy [2]. In this study, we confirmed the protective effect of BBM on hind limb ischemic injury, which is derived from its anti-inflammatory properties. The mechanism of action may be closely related to the inhibition of NF-κB activation.

Previous transcriptomic sequencing studies have found that the NF-κB signaling pathway is significantly activated in the ischemic muscle tissue of the lower limbs [18]. NF-κB is a family of inducible transcription factors that regulate a series of immune responses [24]. BBM is recognized as a natural inhibitor of NF-κB. Previous research has indicated that BBM can treat leukemia by inhibiting the phosphorylation of CaMKII γ, which subsequently prevents the nuclear translocation of NF-κB [10]. Beyond its impact on leukemia, BBM also mitigates liver damage, inhibits osteoclast differentiation, and represses breast cancer growth by targeting the NF-κB signaling pathway [13,17,25]. In this study, we found that BBM can inhibit the increase in NF-κB expression induced by ischemic hind limbs, thereby alleviating tissue inflammation.

Inflammation plays a significant role in the development and progression of peripheral arterial disease [2,18,21,22,26]. The expression of NF-κB, IL-1, and TNF-α signaling pathways is significantly increased in the lower limb muscles of PAD patients [18]. Macrophage M1 phenotype is a pro-inflammatory phenotype, and M1 macrophages secrete a large number of inflammatory factors, promoting local tissue inflammatory response [23]. Activation of the NF-κB signaling pathway promotes the polarization of macrophages to the M1 phenotype [27]. In this study, we found that BBM can inhibit the activation of the NF-κB signaling pathway in the ischemic hind limb muscle tissue, suppress the polarization of macrophages to the M1 phenotype, and decrease the expression level of inflammatory molecules in the tissue. Additionally, an increase in M2-like macrophages enhances VEGFA secretion, fostering angiogenesis and aiding in the repair of ischemic tissue. These results suggest that BBM alleviates tissue inflammation induced by hind limb ischemia by inhibiting the NF-κB signaling pathway.

## 4. Materials and Methods

### 4.1. Preparation of Berbamine

BBM, sourced from MCE (Shanghai, China), was verified to have a purity exceeding 99%. It was prepared for experimental use by dissolving in 1% dimethyl sulfoxide and then further diluting with Phosphate-Buffered Saline (PBS). The dosage of BBM was determined in line with findings from previous research [13].

### 4.2. Animals

Adult male C57BL/6 mice, aged 8 weeks and weighing 25.0 ± 3.0 g, were provided with sterile water and standard rodent chow. These mice were housed in pathogen-free cages supplied by the Model Animal Research Center at Nanjing University. All experimental procedures were conducted in accordance with the guidelines set forth by the Animal Investigation Ethics Committee of Nanjing University and adhered to the regulations outlined in the National Institutes of Health (NIH Publication No. 85–23, revised 1996).

### 4.3. Hindlimb Ischemia Model

In this study, 8-week-old male C57BL/6J mice were subjected to a surgical procedure involving the ligation and transection of the unilateral hind limb femoral artery. The mice were fasted for 8 h preoperatively, and the anesthetic dosage was determined based on their body weight. Anesthesia was administered via an intraperitoneal injection of tribromoethanol at a concentration of 250 mg/kg. The mice were positioned supine on a sterile surgical board and secured with medical tape. The surgical area was then sterilized using iodophors. A longitudinal incision of about 1 cm was made along the blood vessel’s path from the inguinal region to the inner thigh. With the aid of a dissecting microscope, the femoral artery sheath was dissected bluntly, and a suture was placed underneath the sheath for subsequent use. The membranous vascular sheath was carefully punctured with micro-forceps to reveal the femoral artery, femoral vein, and their branches. The femoral artery was then isolated and manipulated using micro-forceps. It was ligated and transected at both the proximal and distal ends with 7–0 surgical sutures. The skin was closed with sutures, and the mouse was placed on a warming pad to recover from the anesthesia. We provided postoperative analgesics: Buprenorphine (0.1 mg/kg body weight, every 8 h) for 2 days.

### 4.4. Drug Treatments and Experimental Design

A total of 36 mice were randomly allocated into 3 groups, with 12 mice in each group: the Sham group, the hindlimb ischemia group, and two ischemia + BBM groups receiving 30 mg/kg of BBM. Mice in the ischemia and ischemia + BBM groups underwent hindlimb ischemia, whereas those in the Sham group did not. After the onset of hindlimb ischemia, mice in the ischemia + BBM groups were administered daily oral gavage of BBM. Twenty-one days later, the mice were euthanized using carbon dioxide, and the muscle tissues were then preserved in an environmental-friendly GD muscle fixative solution for subsequent histological evaluation. Muscle homogenates were utilized to measure the expression levels of specific proteins. Muscle tissue samples from the gastrocnemius muscle were harvested and stored at −80 °C for subsequent analysis.

### 4.5. Histological Evaluation

Samples were immersed in environmentally friendly GD muscle fixative solution and stored at 4 °C for 48 h. Transverse and longitudinal sections, each 3 μm thick, were sliced from the embedded tissue blocks using a microtome. These sections were then subjected to staining with hematoxylin and eosin to assess inflammatory infiltration and interstitial edema.

### 4.6. TUNEL Staining

The TUNEL assay is utilized to detect DNA fragmentation by marking the 3′-hydroxyl ends of double-stranded DNA breaks that occur during apoptosis. It is a common technique for identifying and quantifying apoptotic cells. Following the preparation of tissue samples, which involved paraffin embedding and fixation with paraformaldehyde, staining was carried out using a Cell Apoptosis Detection Kit from Servicebio (Wuhan, China). The number of TUNEL-positive cells was then analyzed using ImageJ software (1.54k).

### 4.7. Immunofluorescence (IF) Assays and Immunohistochemical (IHC) Staining

For IF staining, tissue sections were initially dewaxed and subjected to antigen retrieval and BSA blocking. They were then incubated with the primary antibody, p65 (Proteintech, 10745-1-AP, 1:1000; Manchester, UK), CD86 (Abcam, ab239075, 1:100; Cambridge, UK), CD163 (Proteintech, 16646-1-AP, 1:250) overnight at 4 °C. Afterward, the sections underwent three washes with PBS. Subsequently, the sections were incubated with a fluorophore-conjugated secondary antibody at room temperature for 1h. DNA was stained with DAPI for visualization. Representative areas were imaged using a laser-scanning confocal microscope.

For IHC staining, tissue sections were used to stain CD31 (1:500, Cat 550274) and the secondary antibodies from Santa Cruz Biotech (Santa Cruz, 1:200; Shanghai, China). Images were captured under a light microscope (Zeiss, Jena, Germany). The intensity of positive staining was analyzed by ImageJ software.

### 4.8. Preparation of Cytoplasmic and Nuclear Proteins and Western Blot Analysis

Cytoplasmic and nuclear extracts were prepared using a Nuclear and Cytoplasmic Protein Extraction Kit (Beyotime, Shanghai, China) following the manufacturer’s instructions. Cytoplasmic and nuclear proteins were used for Western blot analysis.

Mouse tissues were homogenized and lysed with RIPA lysis buffer (Beyotime, Shanghai, China). Protein concentration was determined using Bradford buffer. Total protein (20–40 µg/lane) was separated on SDS–PAGE gels and transferred to nitrocellulose membranes. The membranes were blocked with 5% non-fat milk and incubated with primary and secondary antibodies for protein detection. The target bands were detected with the following antibodies: anti-cleaved caspase-3 (Proteintech, 68773-1-Ig, 1:1000), anti-cleaved PARP1 (Hubio, SU0314, 1:1000; Toronto, ON, Canada), anti-p65 (Proteintech, 10745-1-AP, 1:1000), anti-β-Actin (Bioworld, AP0060, 1:10,000; St. Helier, UK), anti-Gapdh (Biodragon, B1030, 1:1000; Szczęsne, Poland), and anti-Histone 3 (Abcam, ab1791, 1:2000). The blots were then incubated with HRP-conjugated secondary antibodies (anti-rabbit or mouse). Protein bands were visualized using enhanced chemiluminescence detection reagents. Relative changes in protein expression were calculated from the mean pixel density using ImageJ and normalized to β-Actin or Gapdh.

### 4.9. Quantitative Real-Time PCR

Total RNA was extracted from tissues or cells using the RNAiso Total RNA Extraction Reagent from Vazyme (Nanjing, China), following the manufacturer’s instructions. Reverse transcription was carried out using the HiScript II 1st Strand cDNA Synthesis Kit from Vazyme, Nanjing, China. qPCR analysis was performed with the ChamQ Universal SYBR qPCR Master Mix from Vazyme, Nanjing, China, on the Applied Biosystems 7300 instrument. β-actin expression served as the endogenous control. The 2^−ΔΔCT^ method was employed to analyze relative fold changes. The primer sequences used were as follows: for β-Actin, forward 5′-GCCACTGCCGCATCCTCTTC-3′ and reverse 5′-AGCCTCAGGGCATCGGAACC-3′; for il10, forward 5′-GCATGGCCCAGAAATCAAGG-3′ and reverse 5′-AATCGATGACAGCGCCTCAG-3′; for Asc, forward 5′-TGCTTAGAGACATGGGCTTAC-3′ and reverse 5′-CTGTCCTTCAGTCAGCACACT-3′; for Caspase-1, forward 5′-GACAAGGCACGGGACCTATGT-3′ and reverse 5′-CAGTCAGTCCTGGAAATGTGC-3′; for IL-18, forward 5′-GACTCTTGCGTCAACTTCAAGG-3′ and reverse 5′-CAGGCTGTCTTTTGTCAACGA-3′; for IL-1β, forward 5′-CAGGCAGGCAGTATCACTCA-3′ and reverse 5′-AGGCCACAGGTATTTTGTCG-3′; for Tnf-α, forward 5′-ACGGCATGGATCTCAAAGAC-3′ and reverse 5′-GGTCACTGTCCCAGCATCTT-3′; and for VEGFA, forward 5′-GGCTCTTCTCGCTCCGTAGT-3′ and reverse 5′-TCACCGCCTTGGCTTGTCAC-3′.

### 4.10. Isolation of Peritoneal Macrophages

To isolate peritoneal macrophages, mice were injected intraperitoneally with 4% starch broth (comprising NaCl 0.5 g, beef extract 0.3 g, peptone 1.0 g, and starch 3.0 g in 100 mL of distilled water) three days prior to euthanasia. Following anesthesia, the abdominal skin was carefully incised by 1 cm, and 5–8 mL of PBS containing 3% FBS was injected into the peritoneal cavity. After massaging for 10 min, the extract was centrifuged at 400× *g* for 5 min. Subsequently, the sediment was plated onto culture dishes containing complete medium. After the cells had adhered, the supernatant was collected 24 h after treatment with BBM 10 μΜ or DMSO in a hypoxic environment.

### 4.11. Angiogenesis Assay

Human umbilical vein endothelial cells (HUVECs) were purchased from Guangzhou Cellcook Company, accompanied by a STR analysis report. Dulbecco’s modified eagle medium (DMEM) supplemented with 10% fetal bovine serum was obtained from Gibco, London, UK. After being treated with the conditioned medium obtained from the peritoneal macrophages as described above for 48 h, the HUVECs were subjected to subsequent experiments. The day before the experiment, the matrigel was placed in an ice box and stored in a 4 °C refrigerator to slowly thaw overnight. Note: prepare 4 °C pre-cooled pipette tips for aspirating the matrigel and a 96-well cell culture plate. Keep the matrigel in the ice box, add 50–100 μL of matrigel to each well of the 96-well plate, aspirate slowly, and add it to the wall to avoid bubble formation. Each well of the 96-well plate was then inoculated with 50 μL of HUVECs at a density of 6000–80,000 cells per well. Be careful not to puncture the matrigel with the pipette tip. Gently shake the culture plate to ensure even distribution of the cells, and then place it in a 37 °C-cell culture incubator. Typically, 6–8 h later, distinct tubular structures can be observed. Images were captured using a microscope and analyzed with Image J software.

### 4.12. Edu Assay

The Edu assay is based on the incorporation of EdU (5-ethynyl-2′-deoxyuridine), a thymidine analog, during DNA synthesis, followed by a click reaction that labels EdU with Alexa Fluor 594 for detection. The HUVECs treated with the conditioned medium as previously described were subjected to the Edu assay using the Edu detection kit (Beyotime, Shanghai, China), following the specific experimental procedure. Images were captured using a fluorescence microscope and analyzed with Image J software.

### 4.13. Statistics

All data are expressed as mean ± SEM. Statistical analysis was conducted using one-way analysis of variance (ANOVA), followed by Tukey’s multiple comparisons test, with GraphPad Prism 8.4.2 software. A *p* value less than 0.05 was deemed statistically significant. * *p* < 0.05, ** *p* < 0.01, *** *p* < 0.001, **** *p* < 0.0001.

## 5. Conclusions

In summary, our findings suggest that BBM exerts a protective effect against hindlimb injury in mice. The underlying mechanism may be intimately linked to the inhibition of NF-κB-mediated inflammation. Consequently, these results suggest that BBM’s protective role in this hindlimb model is significant, and it may alleviate clinical symptoms in patients by inhibiting chronic inflammation caused by lower limb ischemia. However, further clinical trials are needed to confirm these findings.

## Data Availability

The original contributions presented in the study are included in the article, further inquiries can be directed to the corresponding author.

## References

[1] Conte M.S., Bradbury A.W., Kolh P., White J.V., Dick F., Fitridge R., Mills J.L., Ricco J.B., Suresh K.R., Murad M.H. (2019). Global Vascular Guidelines on the Management of Chronic Limb-Threatening Ischemia. Eur. J. Vasc. Endovasc. Surg..

[2] Golledge J. (2022). Update on the pathophysiology and medical treatment of peripheral artery disease. Nat. Rev. Cardiol..

[3] Farber A., Menard M.T., Conte M.S., Kaufman J.A., Powell R.J., Choudhry N.K., Hamza T.H., Assmann S.F., Creager M.A., Cziraky M.J. (2022). Surgery or Endovascular Therapy for Chronic Limb-Threatening Ischemia. N. Engl. J. Med..

[4] Furubeppu H., Ito T., Kakuuchi M., Yasuda T., Kamikokuryo C., Yamada S., Maruyama I., Kakihana Y. (2021). Differential Regulation of Damage-Associated Molecular Pattern Release in a Mouse Model of Skeletal Muscle Ischemia/Reperfusion Injury. Front. Immunol..

[5] Bihari A., Cepinskas G., Forbes T.L., Potter R.F., Lawendy A.R. (2017). Systemic application of carbon monoxide-releasing molecule 3 protects skeletal muscle from ischemia-reperfusion injury. J. Vasc. Surg..

[6] Schiff P.L. (1991). Bisbenzylisoquinoline Alkaloids. J. Nat. Prod..

[7] Liang Y., Xu R.Z., Zhang L., Zhao X.Y. (2009). Berbamine, a novel nuclear factor kappa B inhibitor, inhibits growth and induces apoptosis in human myeloma cells. Acta Pharmacol. Sin..

[8] Zhao W., Jiang Y., Jia X., Wang X., Guo Y. (2023). Berbamine Inhibits the Biological Activities of Prostate Cancer Cells by Modulating the ROS/NF-κB Axis. Anticancer Agents Med. Chem..

[9] Yin L., Zhang L., Luo L., Liu Y., Wang F., Feng Y., Wang H., Han Y., Yan Y., Huang C. (2022). Berbamine reduces body weight via suppression of small GTPase Rab8a activity and activation of paraventricular hypothalamic neurons in obese mice. Eur. J. Pharmacol..

[10] Gu Y., Chen T., Meng Z., Gan Y., Xu X., Lou G., Li H., Gan X., Zhou H., Tang J. (2012). CaMKII γ, a critical regulator of CML stem/progenitor cells, is a target of the natural product berbamine. Blood.

[11] Ling H., Gray C.B., Zambon A.C., Grimm M., Gu Y., Dalton N., Purcell N.H., Peterson K., Brown J.H. (2013). Ca^2+^/Calmodulin-dependent protein kinase II δ mediates myocardial ischemia/reperfusion injury through nuclear factor-κB. Circ. Res..

[12] Liu L., Liang D., Zheng Q., Zhao M., Lv R., Tang J., Chen N. (2023). Berbamine dihydrochloride suppresses the progression of colorectal cancer via RTKs/Akt axis. J. Ethnopharmacol..

[13] Kathem S.H., Abdulsahib W.K., Zalzala M.H. (2022). Berbamine and thymoquinone exert protective effects against immune-mediated liver injury via NF-κB dependent pathway. Front. Vet. Sci..

[14] Yin J.-y., Ye J., Jia W. (2012). Effects and mechanisms of berberine in diabetes treatment. Acta Pharm. Sin. B.

[15] Zhu J.R., Lu H.D., Guo C., Fang W.R., Zhao H.D., Zhou J.S., Wang F., Zhao Y.L., Li Y.M., Zhang Y.D. (2018). Berberine attenuates ischemia-reperfusion injury through inhibiting HMGB1 release and NF-κB nuclear translocation. Acta Pharmacol. Sin..

[16] Zheng Y., Gu S., Li X., Tan J., Liu S., Jiang Y., Zhang C., Gao L., Yang H.T. (2017). Berbamine postconditioning protects the heart from ischemia/reperfusion injury through modulation of autophagy. Cell Death Dis..

[17] Qi G., Jiang Z., Lu W., Li D., Chen W., Yang X., Ding L., Yuan H. (2022). Berbamine inhibits RANKL- and M-CSF-mediated osteoclastogenesis and alleviates ovariectomy-induced bone loss. Front. Pharmacol..

[18] Ferrucci L., Candia J., Ubaida-Mohien C., Lyashkov A., Banskota N., Leeuwenburgh C., Wohlgemuth S., Guralnik J.M., Kaileh M., Zhang D. (2023). Transcriptomic and Proteomic of Gastrocnemius Muscle in Peripheral Artery Disease. Circ. Res..

[19] Sciorati C., Clementi E., Manfredi A.A., Rovere-Querini P. (2015). Fat deposition and accumulation in the damaged and inflamed skeletal muscle: Cellular and molecular players. Cell. Mol. Life Sci..

[20] Signorelli S.S., Katsiki N. (2018). Oxidative Stress and Inflammation: Their Role in the Pathogenesis of Peripheral Artery Disease with or Without Type 2 Diabetes Mellitus. Curr. Vasc. Pharmacol..

[21] Ferreira J., Carneiro A., Vila I., Silva C., Cunha C., Longatto-Filho A., Mesquita A., Cotter J., Mansilha A., Correia-Neves M. (2023). Inflammation and Loss of Skeletal Muscle Mass in Chronic Limb Threatening Ischemia. Ann. Vasc. Surg..

[22] Ferreira J., Roque S., Lima Carneiro A., Longatto-Filho A., Vila I.N., Cunha C., Silva C., Mesquita A., Cotter J., Correia-Neves M. (2024). Reversion of the Inflammatory Markers in Patients With Chronic Limb-Threatening Ischemia. J. Am. Heart Assoc..

[23] Wang N., Liang H., Zen K. (2014). Molecular mechanisms that influence the macrophage m1-m2 polarization balance. Front. Immunol..

[24] Liu T., Zhang L., Joo D., Sun S.-C. (2017). NF-κB signaling in inflammation. Signal Transduct. Target. Ther..

[25] Wang S., Liu Q., Zhang Y., Liu K., Yu P., Liu K., Luan J., Duan H., Lu Z., Wang F. (2009). Suppression of growth, migration and invasion of highly-metastatic hum an breast cancer cells by berbamine and its molecular mechanisms of action. Mol. Cancer.

[26] Signorelli S.S., Fiore V., Malaponte G. (2014). Inflammation and peripheral arterial disease: The value of circulating biomarkers (Review). Int. J. Mol. Med..

[27] Sato S., Sanjo H., Takeda K., Ninomiya-Tsuji J., Yamamoto M., Kawai T., Matsumoto K., Takeuchi O., Akira S. (2005). Essential function for the kinase TAK1 in innate and adaptive immune responses. Nat. Immunol..

